# Mono- and Co-Doped Mn-Doped CsPbCl_3_ Perovskites with Enhanced Doping Efficiency and Photoluminescent Performance

**DOI:** 10.3390/ma16165545

**Published:** 2023-08-09

**Authors:** Hao Jiang, Yiting Zhao, Fangchao Liu, Yongqi Yan, Yinuo Ma, Hexin Bao, Zhongchen Wu, Wei-Yan Cong, Ying-Bo Lu

**Affiliations:** 1 School of Space Science and Physics, Shandong University, Weihai 264209, China; ginger@mail.sdu.edu.cn (H.J.); 202117749@mail.sdu.edu.cn (Y.Z.); 202137781@mail.sdu.edu.cn (F.L.); 202000830030@mail.sdu.edu.cn (Y.Y.); 202000830101@mail.sdu.edu.cn (Y.M.); 202100830002@mail.sdu.edu.cn (H.B.); z.c.wu@sdu.edu.cn (Z.W.); cong_wy@sdu.edu.cn (W.-Y.C.); 2 Shandong Key Laboratory of Optical Astronomy and Solar-Terrestrial Environment, Institute of Space Sciences, Shandong University, Weihai 264209, China

**Keywords:** CsPbCl_3_ perovskites, photoelectronic performance, Mn doping

## Abstract

To investigate the effect of Mn and other metal dopants on the photoelectronic performance of CsPbCl_3_ perovskites, we conducted a series of theoretical analyses. Our findings showed that after Mn mono-doping, the CsPbCl_3_ lattice contracted and the bonding strength increased, resulting in a more compact structure of the metal octahedral cage. The relaxation of the metal octahedral cage, along with the Jahn–Teller effect, results in a decrease in lattice strain between the octahedra and a reduction in the energy of the entire lattice due to the deformation of the metal octahedron. These three factors work together to reduce intrinsic defects and enhance the stability and electronic properties of CsPbCl_3_ perovskites. The solubility of the Mn dopant is significantly increased when co-doped with Ni, Fe, and Co dopants, as it compensates for the lattice strain induced by Mn. Doping CsPbCl_3_ perovskites reduces the band gap due to the decreased contributions of 3d orbitals from the dopants. Our analyses have revealed that strengthening the CsPbCl_3_ lattice and reducing intrinsic defects can result in improved stability and PL properties. Moreover, increasing Mn solubility and decreasing the bandgap can enhance the PLQY of orange luminescence in CsPbCl_3_ perovskites. These findings offer valuable insights for the development of effective strategies to enhance the photoelectronic properties of these materials.

## 1. Introduction

In 2009, Kojima et al. were the first to successfully apply MAPbI_3_ (MA = CH_3_NH_3_) perovskites in the field of solar cells [1]. Since then, the power conversion efficiency (PCE) of perovskite solar cells has impressively increased within only ten years [2,3,4,5,6,7]. Due to their excellent optoelectronic performance, halide perovskites have garnered extensive attention in various optoelectronic fields, such as those examining light emitting diodes (LED) devices and detectors [8,9]. However, the photoluminescence (PL) performance and stability of perovskite materials deteriorate in environments with trace amounts of water and oxygen [10].

Various methods have been employed to enhance the performance of halide perovskites, such as altering the growth technology and modulating the device structure [6,11,12,13,14,15,16]. Doping metal impurities has been confirmed as an effective strategy to modify the physical properties of materials [17,18,19,20,21,22,23,24,25,26,27]. Moreover, doping techniques involving ions like Cu^2+^ and Zn^2+^ have been extensively studied [26,27]. Additionally, there have been significant advancements in the calculation analysis and explanation of these techniques, leading to a more detailed and comprehensive understanding. For instance, Saidaminov et al. added Cd ions to the lattice of FAPbI_3_ (FA = (HC(NH_2_)^2+^)) and found that this significantly reduces the intrinsic vacancies, thereby improving the stability of these mixed perovskites [28]. Similarly, Bi et al. increased the photoluminescence quantum yield (PLQY) of CsPb(Br/Cl)_3_ from 23% to more than 80% via Cu-doping, which is the highest recorded PLQY in mixed halide perovskites at that time [29].

Since the introduction of Mn ions into halide perovskites by Liu et al. in 2016, there has been promising progress in the application of Mn-doped perovskites in LED technology [30]. In 2018, Parobek et al. conducted a study where they prepared Mn-doped CsPbBr_3_ perovskites through direct thermal injection. The study found that the photoluminescence quantum yield (PLQY) of CsPbBr_3_ was significantly improved as a result of the Mn doping [31]. Zhou et al. discovered that the addition of Mn to CsPbCl_3_ and CsPb(Cl/Br_3_) perovskites resulted in red light emissions that could be fine-tuned by adjusting the concentration of Mn doping, thereby expanding the colour range [32]. In addition, Wu and colleagues found that Mn doping significantly improved the photoluminescence properties of Cs_2_AgInCl_6_, making it a promising material for LED applications [33].

Mn dopants have a significant impact on the luminescence of halide perovskites. However, the photoluminescence quantum yield (PLQY) of Mn-doped CsPbCl_3_ (Mn@CsPbCl_3_) is limited by a self-purification effect [34,35,36,37,38]. Xing et al. discovered that introducing Ni dopants into Mn-doped CsPbCl_3_ perovskites results in an increase in the solubility of the Mn dopant from 0.11% to 15.25%. This increase ultimately improves the PLQY of the Mn-generated luminescence in CsPbCl_3_, which is an astonishing phenomenon [39,40].

The addition of Fe and Co impurities to CsPbCl_3_ perovskites promotes the solubility of Mn dopants and improves the PL performance [40]. It is unclear why the second dopant has such a significant influence on the first dopant, or why Ni, Fe, and Co dopants perform similarly. Reports suggest that Ni-doping affects the band structure [40], but some PL and absorption spectra show no effect on the bandgap [41]. The internal mechanism behind Mn and Ni co-doping, as well as the other influences induced by Fe and Co dopants, remains unclear. A systematic theoretical study was conducted on the geometric and electronic structures of Mn@CsPbCl_3_ co-doped with Ni, Fe, and Co. The study analysed the variations of bonding interactions and crystalline structures, which are key factors that determine the band structure and physical properties of CsPbCl_3_ perovskites co-doped with Mn and other metal dopants. The results provide a detailed interpretation of the aforementioned factors.

## 2. Calculation Methods

This paragraph describes the methodology used for density functional theory (DFT) calculations in the Vienna ab initio simulation package (VASP) [41,42]. The Perdew–Burke–Ernzerhof (PBE) method with a generalized gradient approximation (GGA) function is employed to handle exchange and correlation effects between electrons [43]. Meanwhile, we use the Heyd–Scuseria–Ernzerhof hybridized function (HSE06) with the shielding parameter 0.25 to achieve more accurate electronic properties [44]. The electron wave functions are obtained using the PAW method [45] with a cut-off energy of 400 eV. The Brillouin zone [46] is integrated using a 4 × 4 × 4 Monk-horst Pack K point. The convergence criteria for the self-consistent iterative process are set to ≤10^−4^ eV/atoms for total energy and 0.01 eV/Å for force. The bond interactions are analysed using LOBSTER code, which considers: (1) 3p for Cl; (2) 6p for Pb; (3) 3d for Mn; (4) 3d for Ni; (5) 3d for Fe; (6) 3d for Co [47].

## 3. Results and Discussions

### 3.1. Geometric Properties

**Figure 1:**
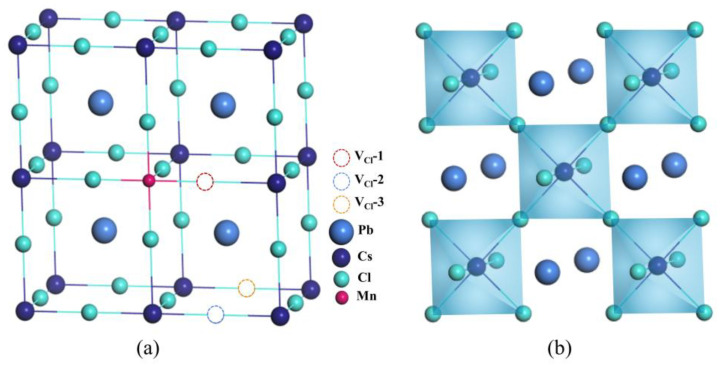
(**a**) Geometric structure of CsPbCl_3_. The constituent elements, the Mn-doping position, and the V_Cl_ positions are denoted accordingly. (**b**) Structure plotted with metal octahedron of CsPbCl_3_.

Figure 1 shows a supercell of CsPbCl_3_ perovskites in cubic phase with a space group of FM-3M and lattice constants of 11.47 Å. Intrinsic defects such as vacancies are commonly found in the lattice of pristine perovskites. These vacancies have a significant impact on the structural stability and optoelectronic properties of the material. Vacancies create carrier traps that impede the transport of electrons, resulting in a reduction of PLQY. In this study, we analyse the distributions of intrinsic vacancies in CsPbCl_3_ perovskites based on previous research. We focus solely on charge neutral defects and calculate the formation energy using the following formula:

(1) $${\;E}_{f}={\;E}_{df}-E_{ini}+\sum_{i}n_{i}\mu_{i}-\sum_{j}n_{j}\mu_{j}$$

*E_df_* and *E_ini_* represent the total energies of the supercells with and without defects, respectively. The number of atoms removed and added during the defect formation process are expressed as *n_i_* and *n_j_*, respectively. *μ_i_* and *μ_j_* are the chemical potentials of elements. The chemical potentials of metal cations and chlorine atoms were calculated from bulk metal materials and gaseous chlorine, respectively. Table 1 lists the calculated formation energies of intrinsic defects, from which we can conclude that the value of Cl vacancy (V_Cl_) is the smallest. This suggests that V_Cl_ is the most preferred intrinsic defect in CsPbCl_3_.

**Figure 2:**
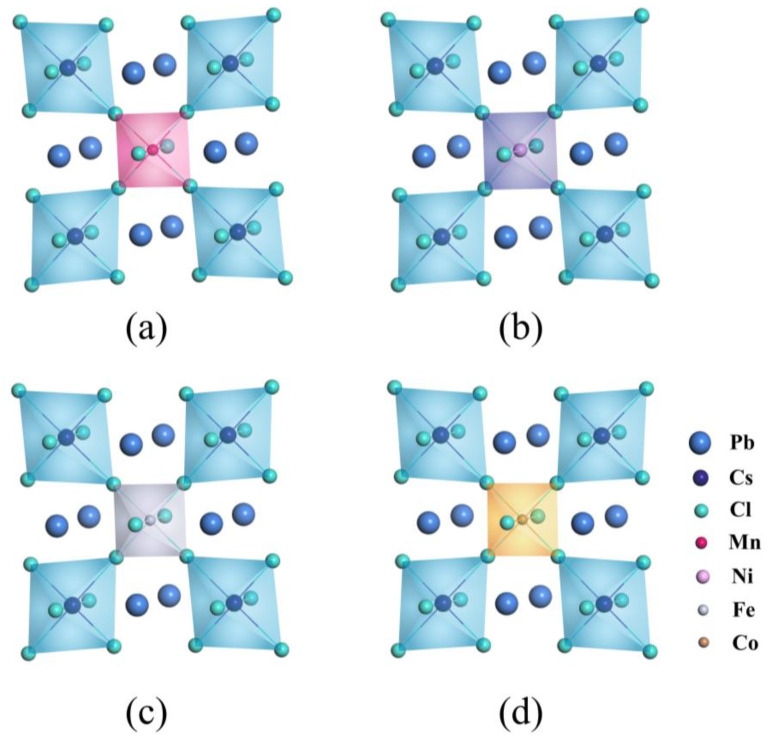
Geometric structures of (**a**) Mn, (**b**) Ni, (**c**) Fe, and (**d**) Co mono-doping CsPbCl_3_.

**Figure 3:**
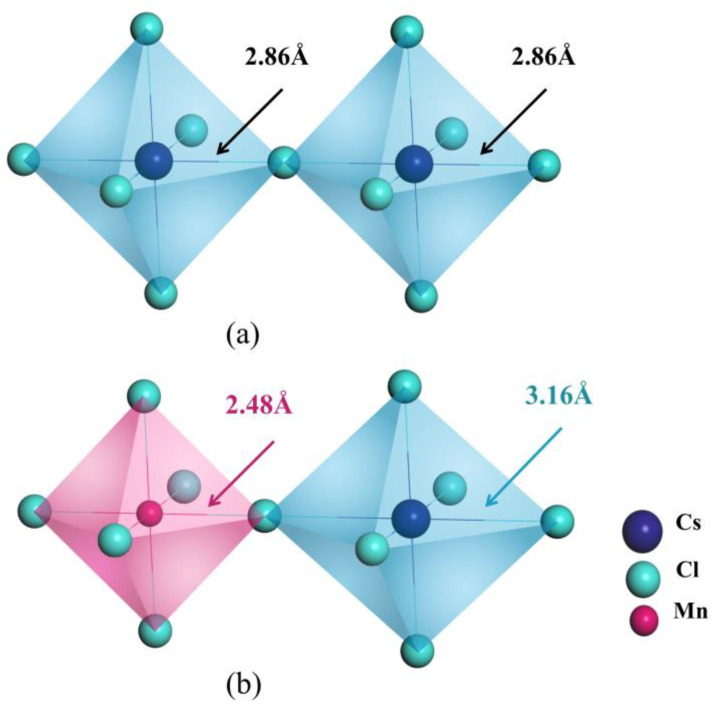
(**a**) Metal octahedron [PbCl_6_] in CsPbCl_3_. (**b**) Contraction of metal octahedron [MnCl_6_] and lattice distortion of [PbCl_6_] in Mn@CsPbCl_3_.

When CsPbCl_3_ perovskites are doped with external impurities like Mn, Ni, Fe, and Co (represented by X), it becomes difficult for X to replace Cl due to the significant difference in electronegativity between X dopants and Cl anions. Additionally, replacing univalent Cs with bivalent X ions introduces a charge imbalance into the crystal. Therefore, defects of X replacing Pb (X_Pb_) are more likely to appear, as shown in Figure 2. For example, as shown in Table 2, when Mn dopant is introduced into the CsPbCl_3_ lattice to form a Mn_Pb_ defect, the lattice contracts due to the smaller radius (81 pm) of Mn^2+^ ions compared to that (133 pm) of Pb^2+^ ions, which is consistent with experimental results. We conducted a detailed analysis of the bond length shrinkage after doping by calculating the data presented in Figure 3 and Table 3. Similar phenomena also occur in CsPbCl_3_ supercells doped with several dopants from the same family as Mn.

The replacement of Pb atoms with X dopants results in a contraction of bond lengths, as shown in Table 2, due to the smaller radii of X compared to Pb. The lattice parameters decrease from 11.47 Å to ~11.28 Å after X-doping. This contraction leads to an increase in bonding strength, making the metal octahedral cage more compact. The denser lattice structure makes it more difficult for atoms to be lost. Additionally, the Jahn–Teller effect suggests that the deformation of the metal octahedron reduces the energy of the entire lattice system. The contraction of the metal octahedral cage reduces lattice strain and stress between atoms, which in turn reduces intrinsic defect and improves the overall stability and electronic properties of CsPbCl_3_ perovskites. Additionally, the Jahn–Teller effect suggests that deformation of the metal octahedron can reduce the energy of the entire lattice system. Overall, these three factors contribute to a decreased likelihood of atom loss in the perovskite’s structure.

To gain a deeper understanding of this intriguing phenomenon caused by the dopant in CsPbCl_3_ perovskites, we assess the stability of a perovskite using the tolerance factor *t*, as described in the Equation (2).

(2) $$t=\frac{R_{A}+R_{X}}{\surd 2(R_{B}+R_{X})}$$

*R_A_*, *R_B_*, and *R_X_* represent the radii of composition ions in ABX_3_ perovskites. The radii used in this study are presented in Table 2. According to theoretical analysis, perovskites can maintain stability when the value of *t* falls within the range of 0.77–1.10. When *t* approaches one, the stability of the perovskite structure is further enhanced. By referring to Table 2 and utilizing Formula (2), the tolerance factor of CsPbCl_3_ is determined to be 0.87. However, when calculating *t* using the radius of the Mn^2+^ ion instead of the original Pb^2+^ ion, *t* is found to be 1.05. Similarly, the calculated *t* values for Ni, Fe, and Co doping are 1.07, 1.08, and 1.06, respectively. These values indicate that they are closer to one compared to the case of undoping, suggesting that the frameworks in the CsPbCl_3_ lattice, which consist of metal-halogen octahedra, are strengthened. Therefore, it can be inferred from this perspective that the overall structural stability of the perovskite crystal is enhanced after doping.

**Figure 4:**
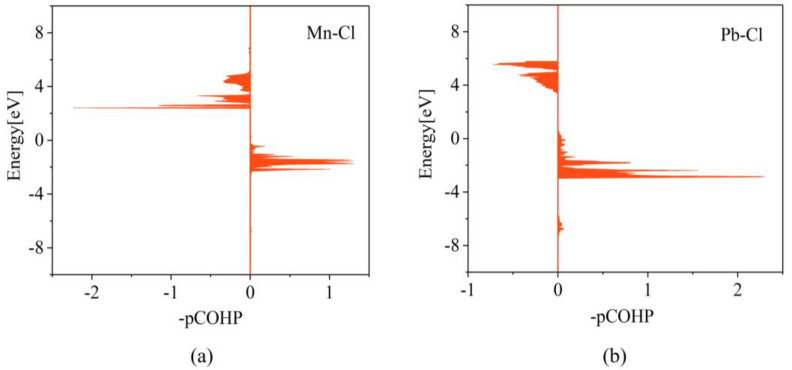
Integral projection COHP diagrams of (**a**) Mn-Cl bond and (**b**) Pb-Cl bond in CsPbCl_3_ systems.

To analyse the variation of bond strength, a Crystal Orbital Hamiltonian Population (COHP) simulation can be used. Figure 4 provides the results of the calculation of integral projection COHP (IpCOHP), which shows that the Mn-Cl bond is stronger than the original Pb-Cl bond, resulting in a slight decrease in bond length. This explains why the CsPbCl_3_ perovskite lattice shrinks and the energy required for defect formation increases. Further analysis is conducted on the variation of orbital bonding induced by different dopant atoms including Fe, Co, and Ni. Table 3 and Figure S1 display the results of the IpCOHP calculation. It can be observed that the metal-chlorine bonds in the three different doping configurations are enhanced to varying degrees. Specifically, the IpCOHPs of the Ni-Cl bond, Co-Cl bond, Fe-Cl bond, and Mn-Cl bond are larger than those of Pb-Cl bond. The bonding strength follows the order of Ni-Cl bond < Co-Cl bond < Fe-Cl bond < Mn-Cl bond. This order is consistent with the defect formation energies and bond lengths, which further confirms our theoretical explanation for the metal octahedral contraction. Additionally, it was found that the peak bonding energies of Mn-Cl, Ni-Cl, Fe-Cl, and Co-Cl are significantly lower than that of Pb-Cl bond. This reduction in energy makes the perovskite systems more stable and strengthens the crystal structures.

To investigate the impact of X dopants on the pristine CsPbCl_3_ systems with intrinsic defects, we constructed supercells containing X_Pb_ external defects and the most common intrinsic V_Cl_ defect. The relative position between the two defects is crucial and requires careful investigation. We considered three configurations: nearest neighbouring, next nearest neighbouring, and farthest positions from the X_Pb_ defects. Figure 1a shows the positions of V_Cl_ defects in these configurations, which are indicated by dashed circles labelled as V_Cl_-1, V_Cl_-2, and V_Cl_-3, and Table 2 presents the three different defects as V_Cl_^1^, V_Cl_^2^, and V_Cl_^3^ respectively. The results of the formation energy calculations indicate that the configuration containing the nearest neighbouring X_Pb_V_Cl_-1 complex has the lowest formation energy, regardless of the dopant used (X = Mn, Ni, Fe, Co). This suggests that X_Pb_V_Cl_-1 is the most energetically favourable. Table 2 reveals that the X^2+^ ions have smaller radii than Pb^2+^ ions, resulting in shorter X-Cl bond lengths compared to the original Pb-X bond. For example, in the case of Mn-doping, the Mn-Cl bond is reduced to a length of 2.48 Å, causing high stress in the CsPbCl_3_ lattice. This stress leads to noticeable lattice distortion near the [MnCl_6_] octahedron, which is depicted in Figure 3. The elongation of the Pb-Cl bond length from 2.86 Å to 3.16 Å is caused by the neighbouring [PbCl_6_] octahedron sharing a joint chloride ion, which causes a strong stretch in the [MnCl_6_] octahedron. However, this stress can be effectively released by the nearest V_Cl_ vacancy.

Previous studies have shown that it is challenging to dope Mn to a high concentration due to its self-purification mechanism [35,38,48]. However, some reports suggest that co-doping Mn with other impurities can overcome this limitation and enhance the physical properties induced by the Mn dopant, such as the PL performances [40]. In this study, we analyse the Mn-Ni co-doping system to examine these phenomena.

**Figure 5:**
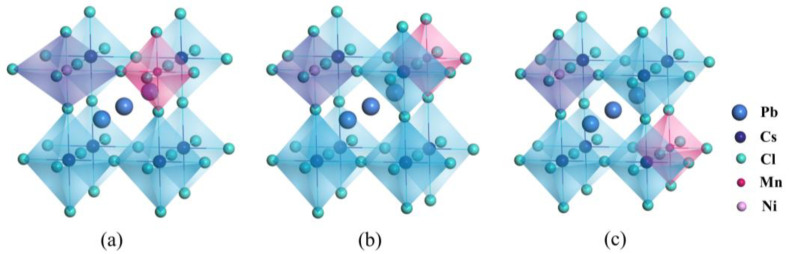
Configurations of (**a**) co#1, (**b**) co#2, and (**c**) co#3 for Mn and Ni co-doping CsPbCl_3_ perovskites.

To study the influence of the distance between Mn and the extra metal dopant on crystal structures and defect formation energies, we constructed three configurations for each co-doping strategy, namely co#1, co#2, and co#3, where the distance between Mn and the other metal dopant varied from near to far. Figure 5 illustrates these distributions. For Ni mono-doped and co-doped with Mn, we evaluated the solubility of the Mn dopant in CsPbCl_3_ perovskites by using the defect formation energies of the Mn_Pb_ defect.

Table 1 shows that when Mn is mono-doped into CsPbCl_3_ perovskites, the formation energy of the Mn_Pb_ defect is 3.96 eV. However, when Mn is co-doped with Ni dopant, the formation energy of the Mn_Pb_ defect decreases significantly, indicating an improvement in the solubility of the Mn dopant in CsPbCl_3_ perovskites. The configuration with the Mn dopant occupying the nearest neighbouring lattice site to the extra Ni dopant consistently results in the lowest formation energy. In other words, Mn and Ni dopants tend to co-appear in pairs. Incorporating mono-doping metal dopants into CsPbCl_3_ perovskites can lead to lattice distortions. However, the distortions can be corrected by the nearest neighbouring metal dopants, as seen in nonlinear molecules with spatially degenerate electronic ground states. This correction reduces the energy by eliminating degeneracy and helps to relax the stress throughout the entire lattice, as illustrated in Figure 5.

### 3.2. Electronic Properties

**Figure 6:**
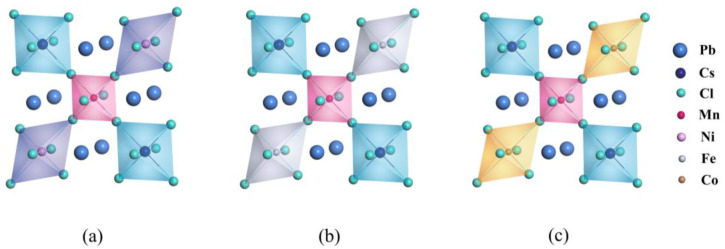
Geometric structures of (**a**) MnNi@CsPbCl_3_, (**b**) MnFe@CsPbCl_3_, and (**c**) MnCo@CsPbCl_3_.

**Figure 7:**
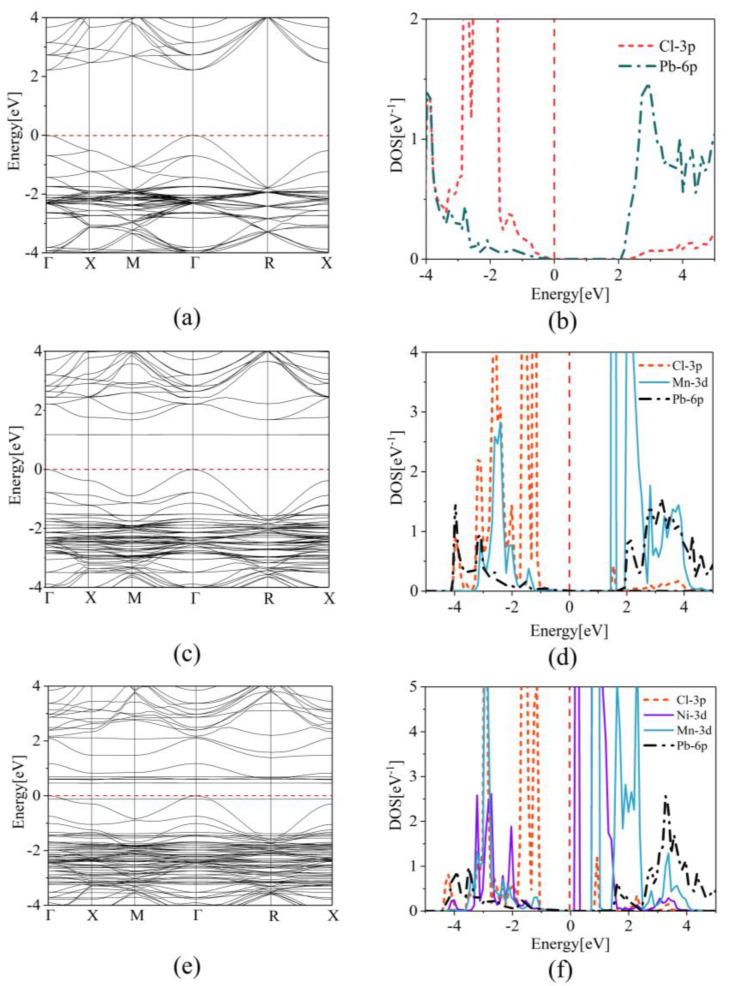
(**a**) Band structure and (**b**) DOS of pristine CsPbCl_3_ perovskites. (**c**) Band structure and (**d**) DOS of Mn mono-doping CsPbCl_3_ perovskites. (**e**) Band structure and (**f**) DOS diagram of Mn and Ni co-doping CsPbCl_3_ perovskites.

**Figure 8:**
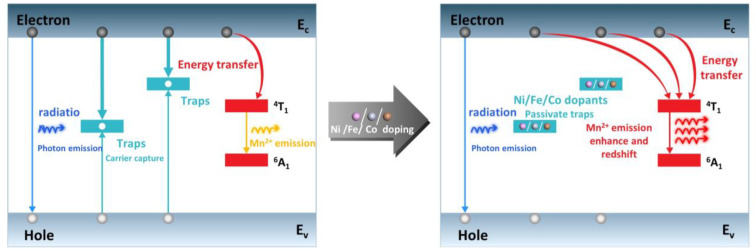
Schematic diagram of Mn-induced luminescence in CsPbCl_3_ perovskites improved by the Ni, Fe, Co co-doping.

To investigate the luminescence properties of CsPbCl_3_ perovskites, we examined the impact of external dopants on the band structures and density of states. Our analysis compared the band gap of undoped cubic CsPbCl_3_, which exhibited a direct band structure with a gap of 2.22 eV, to the band gap after Mn, Ni, Fe, and Co doping, as illustrated in Figure 6. Figure 7 illustrates our findings. Our analysis of the PDOS revealed that the Cl-3p orbital dominates the valence band maximum (VBM) of CsPbCl_3_, while the Pb-6p orbital controls the conduction band minimum (CBM). The band structures of CsPbCl_3_ perovskites with Mn, Ni, Fe, and Co doping are depicted in Figure 8 and Figure S2 in the Supplementary Materials. To ensure the reliability of the data, the results calculated by the HSE method are provided in Figures S5 and S6 in the Supplementary Materials. Upon comparison, it is evident that the changing trend of the energy bandgap remains consistent before and after doping. The figures illustrate that the direct band gap of CsPbCl_3_ perovskites remains unchanged, but the band gaps shift to 1.68 eV, 1.82 eV, 1.38 eV, and 2.16 eV, respectively, due to the introduction of impurity levels. For instance, after Mn-doping, the Mn-3d orbital significantly hybridizes with the band edges. Specifically, the Mn-3d orbital strongly hybridizes with the Pb-6p orbital near CBM, leading to a decrease in the band gap. Similarly, in Ni, Fe, and Co mono-doping CsPbCl_3_ systems, the strong hybridizations of Ni-3d, Fe-3d, and Co-3d with Cl-3p near CBM cause a decrease in the band gaps. The reduction of the band gap in CsPbCl_3_ results in a red shift in its luminescence, which can be advantageous for the production of orange light emitting diodes.

In this study, the electronic properties of MnNi@CsPbCl_3_ were investigated by examining the band structures and density of states of Ni, Fe, and Co co-doping with Mn. The results showed that strong interactions between Mn and Ni resulted in manifest impurity bands, which occupied the nearest neighbouring [XCl_6_] octahedra in CsPbCl_3_ perovskites. The valence band maximum (VBM) was mainly contributed by the hybridization of Mn-3d orbital, Ni-3d orbital, and Cl-3p orbital, while the conduction band minimum (CBM) was dominated by the hybridization of Mn-3d orbital and Ni-3d orbital. This led to a decrease in the band gaps, causing a red shift in the luminescence of perovskites and an enhancement of the overall electrical conductivities. Figure S3 in the Supplementary Materials and Figure 7e, f provides visual representations of the findings.

The doping efficiency and luminescence efficiency of the Mn dopant in CsPbCl_3_ can be improved through co-doping with Ni, Fe, and Co. The difference in radii and electronegativities between Mn and host Pb atoms leads to a self-purification effect in CsPbCl_3_ perovskites, which minimizes the doping concentration of Mn. However, co-doping Mn with Ni, Fe, and Co, whose outermost electronic configurations are similar to that of Mn, can effectively relax the lattice stress and distortion induced by mono-doping Mn. This eliminates intrinsic defects and trap states inside the CsPbCl_3_ lattices. The defect states induced by Ni, Fe, and Co dopants are at a similar level to those of Mn dopant, which means they do not create additional traps for electrons. Therefore, the doping and emission efficiency of the Mn dopant are effectively enhanced, as illustrated in Figure 8.

## 4. Conclusions

This study utilizes first-principles calculations to analyse the geometric and electronic structures of CsPbCl_3_, both mono-doped with Mn and co-doped with Mn, Ni, Fe, and Co. The study thoroughly examines the formation energies, energy bands, DOS, and COHP of various defects in the pristine, mono-doping, and co-doping CsPbCl_3_ systems. The results show that Mn mono-doping CsPbCl_3_ perovskites exhibit a contraction of bond lengths and an increase in bonding strengths, resulting in a more compact metal octahedral cage structure. This contraction reduces the lattice strain and stress between atoms in the octahedron. The Jahn–Teller effect causes deformation of the metal octahedron, which in turn reduces the energy of the entire lattice system. This, combined with the reduction of intrinsic defects and improvement of electronic properties, leads to improved stability of CsPbCl_3_ perovskites. When Mn is co-doped with Ni, Fe, and Co dopants, the lattice strain induced by Mn is compensated for and the solubility of the Mn dopant is significantly increased. Doping Mn into CsPbCl_3_ perovskites, either alone or in combination with other dopants, leads to a noticeable decrease in band gap, primarily due to the contribution of 3d orbitals of the doped atoms. Therefore, it can be concluded that strengthening the CsPbCl_3_ lattice and reducing intrinsic defects are beneficial for improving stability and PL properties between atoms. The increase in Mn solubility and decrease in bandgap contribute to the improvement in the orange luminescence’s PLQY. These findings aid in the understanding of the metal dopant modulation effect in halide perovskites and offer effective strategies for enhancing their photoelectronic properties.

## Figures and Tables

**Table 1:** Formation energies of various defects in CsPbCl_3_ systems.

Doping Strategies	Defect Type	*E_f_* (eV)	Doping Strategies	Defect Type	*E_f_* (eV)
Intrinsic Defects	V_Cl_	3.15	Mono-Doping	Mn_Pb_	3.96
	V_Cs_	3.96		Mn_Pb_V_Cl_^1^	6.83
	V_Pb_	3.96		Mn_Pb_V_Cl_^2^	6.86
Mono-Doping	Ni_Pb_V_Cl_^1^	4.78		Mn_Pb_V_Cl_^3^	6.85
	Ni_Pb_V_Cl_^2^	5.16	Co-Doping	MnNi^1^	2.56
	Ni_Pb_V_Cl_^3^	5.07		MnNi^2^	2.84
	Co_Pb_V_Cl_^1^	5.63		MnNi^3^	3.34
	Co_Pb_V_Cl_^2^	5.93		MnCo^1^	3.17
	Co_Pb_V_Cl_^3^	5.85		MnCo^2^	3.36
	Fe_Pb_V_Cl_^1^	6.06		MnCo^3^	3.58
	Fe_Pb_V_Cl_^2^	6.28		MnFe^1^	3.51
	Fe_Pb_V_Cl_^3^	6.19		MnFe^2^	3.53
				MnFe^3^	3.69

**Table 2:** Crystal radii (CR) and the coordination numbers (CN) of ions in doped-CsPbCl_3_ systems.

Ion	Cs^+^	Cl^−^	Pb^2+^	Mn^2+^	Fe^2+^	Co^2+^	Ni^2+^
CN	12	6	6	6	6	6	6
CR(pm)	202	182	133	81	75	79	77

**Table 3:** The bond lengths (Å) and the corresponding integral projection COHP (eV per cell) in the pristine, mono-, and co-doping CsPbCl_3_ systems.

Systems	Bonds	Bond Lengths	-IpCOHP
CsPbCl_3_	Pb-Cl	2.86	1.78
Ni@CsPbCl_3_	Ni-Cl	2.52	3.52
Co@CsPbCl_3_	Co-Cl	2.46	3.93
Fe@CsPbCl_3_	Fe-Cl	2.45	4.07
Mn@CsPbCl_3_	Mn-Cl	2.48	4.08
MnNi^1^@CsPbCl_3_	Mn-Cl	2.30	4.32
	Ni-Cl	3.42	4.43
MnCo^1^@CsPbCl_3_	Mn-Cl	2.32	4.15
	Co-Cl	3.40	4.82
MnFe^1^@CsPbCl_3_	Mn-Cl	2.35	4.08
	Fe-Cl	3.44	3.89

## Data Availability

Data is unavailable due to privacy.

## Supplementary Materials

The following supporting information can be downloaded at: https://www.mdpi.com/article/10.3390/ma16165545/s1, Figure S1. pCOHP diagrams of (a) Ni-Cl bond, (b) Fe-Cl bond and (c)Co-Cl bond in CsPbCl_3_ systems. Figure S2. (a) Band structure and (b) DOS diagram of Ni-doping CsPbCl_3_. (c) Band structure and (d) DOS of Fe-doping CsPbCl_3_. (e) Band structure and f) DOS of Co-doping CsPbCl_3_. Figure S3. (a) Band structure and (b) DOS diagram of Mn and Fe co-doping CsPbCl_3_. (c) Band structure and (d) DOS diagram of Mn and Co co-doping with CsPbCl_3_. Figure S4. The convergency test of the CsPbCl_3_ systems using different Monkhorst Pack K point meshes. Figure S5. (a) Band structure and (b) DOS of pristine CsPbCl_3_ perovskites. (c) Band structure and (d) DOS of Mn mono-doping CsPbCl_3_ perovskites calculated by the HSE method. Figure S6. (a) Band structure and (b) DOS diagram of Ni-doping CsPbCl_3_. (c) Band structure and (d) DOS of Fe-doping CsPbCl_3_. (e) Band structure and f) DOS of Co-doping CsPbCl_3_ calculated by the HSE method.
