# Supplementary material for: Mono- and Co-Doped Mn-Doped CsPbCl3 Perovskites with Enhanced Doping Efficiency and Photoluminescent Performance
Source: Materials (Basel). 2023 Aug 9;16(16):5545. doi: 10.3390/ma16165545 (PMC10456559; doi:10.3390/ma16165545)
Supplement: Supplementary file 1 [file materials-16-05545-s001.zip › materials-2505497-supplementary.pdf]

materials

Supporting Information

Mono- and Co-Doped Mn-Doped $CsPbCl_3$ Perovskites with Enhanced Doping Efficiency and Photoluminescent Performance

Hao Jiang [1], Yiting Zhao [1], Fangchao Liu [1], Yongqi Yan [1], Yinuo Ma [1], Hexin Bao [1], Zhongchen Wu [1,2], Weiyan Cong [1] and Yingbo Lu [1,*]

[1] School of Space Science and Physics, Shandong University, Weihai 264209, China; ginger@mail.sdu.edu.cn (H.J.); 202117749@mail.sdu.edu.cn (Y.Z.); 202137781@mail.sdu.edu.cn (F.L.); 202000830030@mail.sdu.edu.cn (Y.Y.); 202000830101@mail.sdu.edu.cn (Y.M.); 202100830002@mail.sdu.edu.cn (H.B.); z.c.wu@sdu.edu.cn (Z.W.); cong_wy@sdu.edu.cn (W.-Y.C.)

[2] Shandong Key Laboratory of Optical Astronomy and Solar-Terrestrial Environment, Institute of Space Sciences, Shandong University, Weihai 264209, China

[*] Correspondence: lyb@sdu.edu.cn

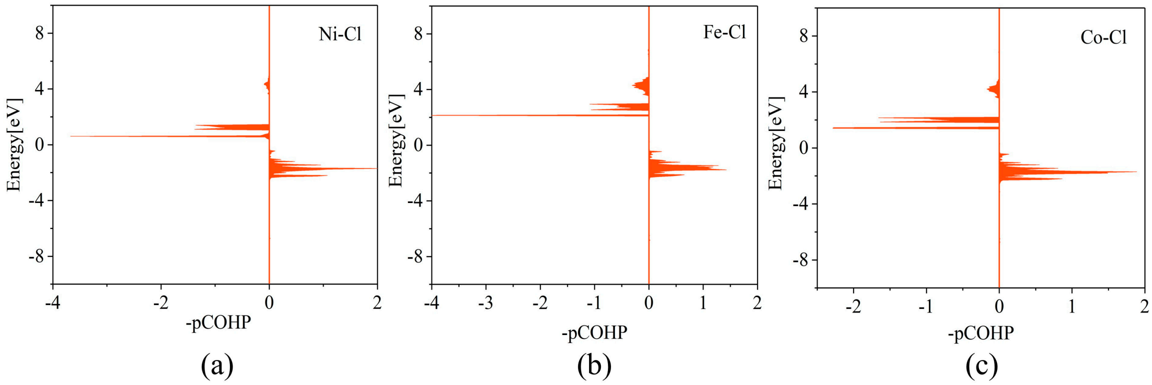

Figure S1. pCOHP diagrams of (a) Ni-Cl bond, (b) Fe-Cl bond and (c)Co-Cl bond in $CsPbCl_3$ systems.

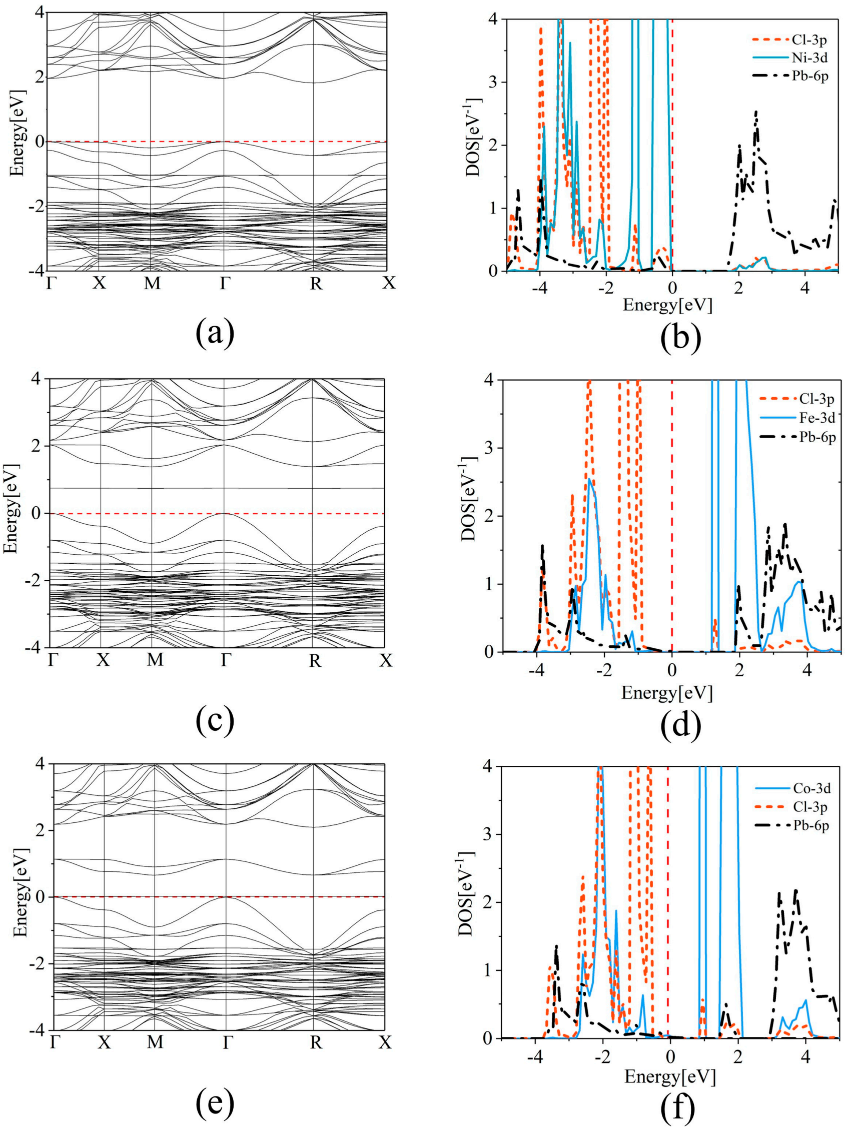

Figure S2. (a) Band structure and (b) DOS diagram of Ni-doping $CsPbCl_3$. (c) Band structure and (d) DOS of Fe-doping $CsPbCl_3$. (e) Band structure and (f) DOS of Co-doping $CsPbCl_3$.

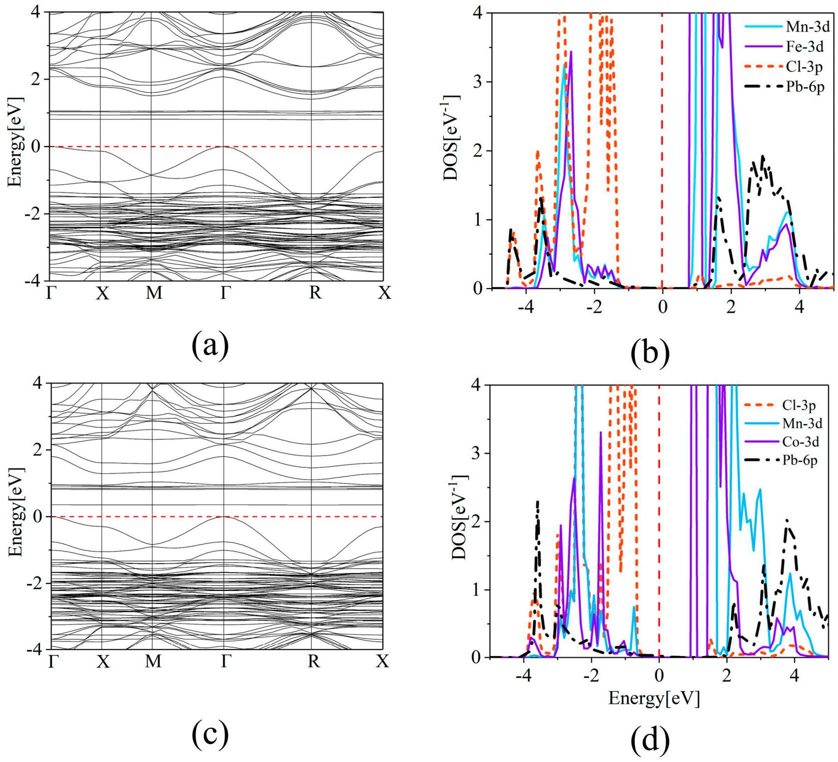

Figure S3. (a) Band structure and (b) DOS diagram of Mn and Fe co-doping $CsPbCl_3$. (c) Band structure and (d) DOS diagram of Mn and Co co-doping with $CsPbCl_3$.

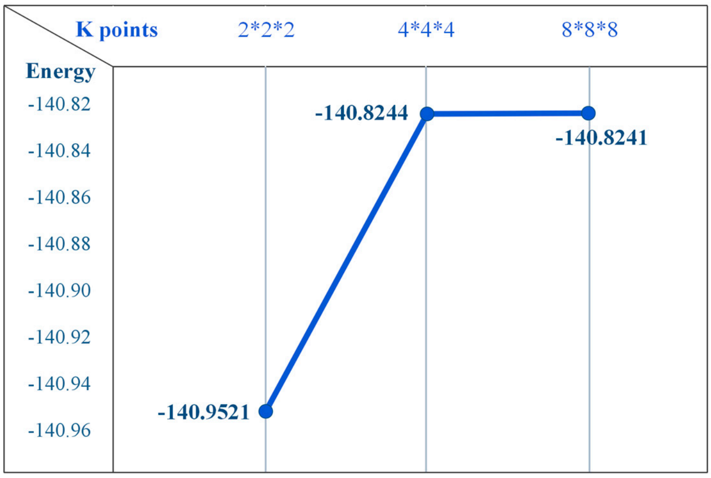

Figure S4. The convergency test of the $CsPbCl_3$ systems using different Monkhorst Pack K point meshes.

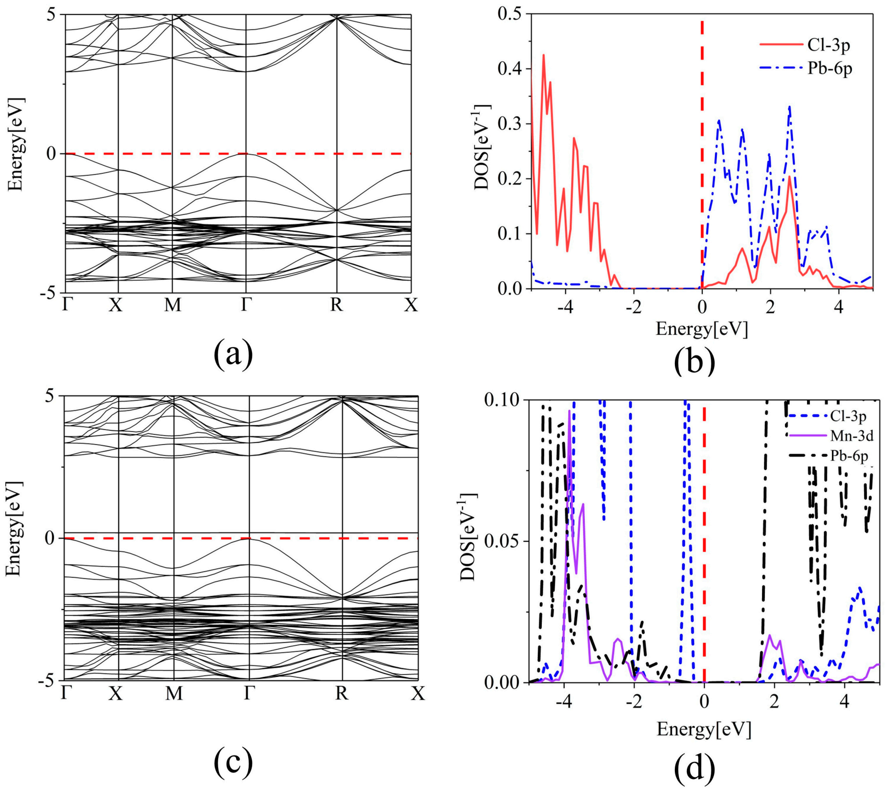

Figure S5. (a) Band structure and (b) DOS of pristine $CsPbCl_3$ perovskites. (c) Band structure and (d) DOS of Mn mono-doping $CsPbCl_3$ perovskites calculated by the HSE method.

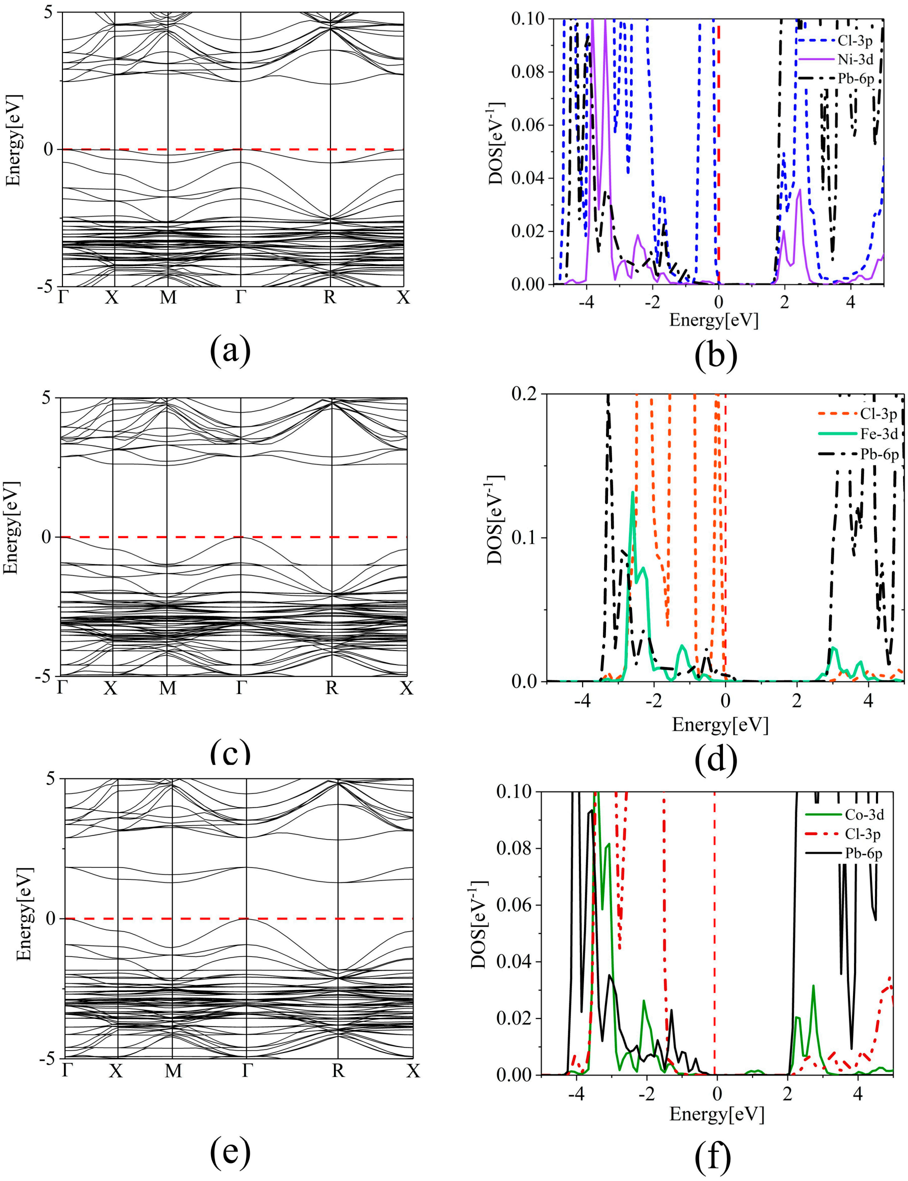

Figure S6. (a) Band structure and (b) DOS diagram of Ni-doping $CsPbCl_3$. (c) Band structure and (d) DOS of Fe-doping $CsPbCl_3$. (e) Band structure and (f) DOS of Co-doping $CsPbCl_3$ calculated by the HSE method.
